# Practical Medicine, Etc.

**Published:** 1857-01

**Authors:** 


### PRACTICAL MEDICINE, ETC.

1. THE MICROSCOPE IN THE DIAGNOSIS OF PHTHISIS.—[In a recent work by Dr. Gustaf von Düben, of Stockholm, entitled, "A Review of the Contributions yielded by the Microscope to Medical Diagnosis," the aid furnished by the microscope in the diagnosis of phthisis is given in the following statement, which we copy from a review of the work in the November No. of the Dublin Quarterly Journal of Medical Science.]

"If the practical physician meets with a patient who describes a previous pneumonia, of which his present illness is a consequence, or in whom the physical signs, in agreement with the history of the case and the rational symptoms, disclose the lesion which is developed in the lungs, he has no doubt as to the diagnosis. But if, on the other hand, he be called to a young patient, in whom all other modes of investigation are merely sufficient to establish the existence of a chronic catarrh, the microscope alone can decide whether this catarrh is uncomplicated, or whether tuberculosis lurks behind it. The honor of the discovery of the microscopic sign belongs to Professor Schroeder van der Kolk, of Utrecht, who published an essay on the subject, which was translated into Swedish by Ehr. Ekströmer, Chir. Magr., and inserted in the Hygeia for 1850, pp. 21–39. Singularly enough, the matter seems to have attracted but little notice, although it is, as I can testify from extensive investigations, of the greatest importance; the positive result has never as yet been disproved by the issue of the case.

"We know from pathological anatomy that in pulmonary catarrh the mucous membrane of the bronchi only is destroyed, and even this imperfectly, whether the disease has an acute or chronic course. The same is the case with acute pneumonia in process of recovery. In none of these cases can we, therefore, *a priori*, expect to find in the sputa any other elementary parts than those belonging to this membrane, and experience has confirmed this view.

"In pneumonia, on the contrary, which assumes a chronic form, or where, through the formation of pus, destruction of parts of the lung takes place, other portions of the bronchi, besides the mucous membrane, are attacked and are destroyed by the ulcerative process. The same occurs in tuberculosis. In both these cases we may beforehand expect, and afterwards find, in the sputa, fragments of the bronchial walls. The characteristic point in the sputa, from vomicæ, is the presence of the elastic filaments of these walls; they are *never* wanting so soon as a progressive vomica is met with.

"So soon as these elastic filaments are found in the sputum, they are certain signs of a vomica. It is, however, only important to seek them in cases where other signs are not sufficient to establish a certain diagnosis. Such a case is incipient tuberculosis.

"We usually find signs of more or less extensive chronic catarrh. The sputa consequently contain a great quantity of pus-cells, and of oval or round epithelium. They have all the external and microscopic characters of concocted sputa. If we now take out of such a sputum, with a forceps, a small portion from one of the clearer parts, we see in the white or whitish-yellow mass, and bring it thinly spread out under the microscope, at a magnifying power of 250, we usually find in it fragments of the elastic tissue, particularly if the tuberculosis is still recent. Experience has, in fact, shown that the elastic tissue is more easily found when the disease has not lasted long, and for this there are two reasons.

"In the first place, in tuberculosis of small extent, the catarrh is more limited to the bronchi coming from the tuberculized part; consequently, the formation of the sputa must take place there, and the latter therefore contain the object of our search in greater quantity; secondly, at the commencement of the ulcerative process, the tissue, being less completely destroyed, must yield large and more distinct portions to the bronchial secretion than afterwards, when a more violent action in the walls of the cavity breaks down the pulmonary tissue more perfectly before it can be separated. We have, therefore, in the sputum of recent tuberculosis, both a less volume to examine, and larger fragments to find—two very great advantages, as the question is precisely to be able to make an early diagnosis."

2. ON THE TREATMENT OF CHOREA BY GYMNASTIC EXERCISES.—By *Dr. Blache.*

M. Blache observes, that two indications should guide us in our treatment of this affection: 1. To restore to the will its empire over the muscular contractions—i. e., regularize the movements; and 2. So to say, reform the constitution of the patients.

M. Blache regarded the methodical use of sulphur baths as the best constitutional remedy for the affection, until he had recourse to gymnastics. The great success which attended the application of these in 1847, under the skilful direction of M. Laisne, to scrofulous subjects at the *Hôpital des Enfans*, induced the directors to erect large gymnasia, and extend their employment to various other diseases, among which was chorea. In the present paper, M. Blache gives an account of 108 cases so treated, 100 being first attacks, and only 8 relapses—an important distinction, as the ordinary duration of a case of chorea is diminished by a number of relapses. These 108 were divided into two categories, according to the severity of the disease; one of these being composed of 34 cases, of mean intensity, and the other of 74, in which the agitation was as violent as possible. The whole of the 34 were cured in a mean period of twenty-six days and eighteen *séances;* of the 74, 68 were cured in forty-five days with thirty-one *séances.* Therefore there remained but 6 cases which may be regarded as failures. There were examples of chronic chorea, which in the end was also cured, requiring 122 days and 63 *séances.* Calculating in another mode, we have 102 cases in 39 days, and 6 in 122 days.

For the description of the procedure that is had recourse to, M. Blache takes an aggravated case, in which the movements are violent and speech impossible. Here the will of the subject being powerless, nothing can be demanded of him; and the gymnastics must be entirely passive. M. Laisne, aided by three or four intelligent pupils, fixes the patient on his back in bed, and retains him thus motionless for ten or fifteen minutes. He then shampoos with the open hand, the chest and limbs for a long time, following this by brisk friction. Similar manipulation is pursued at the back parts of the body, and especially at the nape, and over the muscular masses alongside the spine. A *séance* of this kind lasts about an hour, and is repeated for three or four days in succession. An amendment in the disordered contractions is observed after each; the child evidences its contentment, and calm sleep is restored. The following days, without entirely discontinuing the shampooing, the child is taught to execute very regular and perfectly rhythmical movements. Thus, suppose the arms are hanging in a state of supination by the side of the body, the operator takes hold of them by the wrist, bends the forearm upon the arm, carries the latter directly upward and forward, and then replaces the forearm in a state of extension. The hands are now raised in a parallel manner above the head, and from thence they are brought down to their point of departure, always following a well-marked ternary measure. This manœuvre is executed a great number of times, with much regularity. The inferior extremities are submitted in their turn to analogous movements: the leg is bent rapidly on the thigh, and this on the pelvis, when both are brought into extension, following a binary measure.

It is clear that the manipulations employed must impart remarkable activity to the capillary system of the skin and subjacent tissues, and through this to the intimate process of nutrition. The movements are so combined, that muscles whose motions are synergetical are brought into regular and simultaneous action. Unable to contract spontaneously and with regularity, they seem quite passive, so that the limbs may be bent or extended without the will of the patient contributing to the effect. Indeed, this generally opposes it, and it is only obtainable by employing a certain amount of force. But after one or two *séances*, the hand of the operator is enabled to follow the contractions which come to his aid with regularity. Every day the command of the will over the muscular system is strengthened, the abnormal movements at the same time diminishing in frequency and intensity. Not infrequently, during the first days, pains are excited in some of the joints by movements at all strong; but these, which some have considered of a rheumatic character, disappear after a few *séances*. After the employment of these passive movements for eight or ten days, very marked improvement is observed, for the child can now speak intelligibly, feed itself, and, in some instances, walk about the ward. He now joins the gymnasium, and takes part in the exercises under the surveillance of the master or a monitor-pupil. These exercises are graduated, and have in view the production of regular and easy physiological movements of the trunk and limbs—movements in which the will and the attention are called into play as much as are the physical powers. A great number of the manœuvres are performed in common, and during their execution the master and his pupils sing an air in two or three well-marked times, according as the exercises are performed in binary or ternary measure. The little patients, ranged in groups, are led away by the rhythm and by imitation. Other exercises are individual, and executed by each child according to its strength, all having for their object the rousing the attention, and bringing the muscular contractions under the empire of the will.

The spirit of order and discipline exerts upon these children the most salutary influence. The attention, zeal, and great address of M. Laisne, aided by the means for insuring safety in the gymnasium, have prevented any kind of accident occurring. During the first ten days, the children pursue the exercises with ardor. They are desirous of doing well, and their disposition seems to undergo a favorable change as they become more lively and open, and at the same time more docile. The organic functions are remarkably influenced from the first, the appetite becoming very keen, and requiring a proportionate supply of aliment; the muscular power increasing, and even already some increase of flesh being apparent. From the tenth and twelfth day, this amendment is subjected to some check; and we must now support the will and courage of the patient, and the more so, because the children endowed with most courage, determination, and docility, make the most rapid progress. After some days of this resistance, renewed improvement is observed, and we may be sure that the cure will now prove prompt and radical. Whatever the future may reveal, hitherto, no relapse has been met with.

M. Blache enters into a comparison of the relative efficacy of gymnastics and sulphurous baths; and although employed alone, the former mode of treatment is to be preferred, yet the combination of the two modes is often desirable.—*Mem. Imp. Acad. of Med.—Brit. and Foreign Med. Chir. Review*, Oct., 1856.

3. LARGE INTESTINAL CALCULUS.—[In a recent collection of tinted lithographs, taken from important preparations in the Anatomical Museum at Stockholm, is a representation of a remarkable intestinal concretion; an account of which we extract from the October No. of the British and Foreign Medico-Chirurgical Review.]

The case of intestinal calculus is a rare specimen of the development of an enormous concretion, which formed in the caput cæcum and appendix vermiformis of a laboring man, aged twenty-two. It was passed per anum after intense suffering, the dislodgment having been apparently effected by the use of seal oil, which the patient prescribed for himself, after having been under the hands of medical men to no purpose. The calculus weighed fourteen ounces and a quarter, was nearly seven inches long, and about two broad. Its shape was moulded to the vermiform process and the cæcum, which it had occupied. The surface was granular, exhibiting impressions of the mucous membrane. About the middle of the concretion was a minute cavity, about 0·1 inch (4 millimètres) in diameter, containing a small coagulum, round which the calculous matter was arranged concentrically as far as the surface; the further accession of calculous matter in the direction of the two ends of the concretion also exhibited a generally concentric arrangement round the coagulum, but the circles were necessarily not completed. The layers surrounding the coagulum were alternately tawny and containing a hairy substance, and yellow, more solid and earthy. This alternation was particularly regular for the first six layers, the hairy layers being less broad than the others. The hairs, on microscopic examination, were found to be the hairs that invest the caryopsis of oats.

The following is the chemical analysis of the inner layers:

| | |
|---|---|
| Matters soluble in ether, | 1·58 |
| Soapy matters soluble in pure alcohol, the bases of which were soda, lime, and magnesia, | 0·30 |
| Fatty salts and acids soluble in water, | 5·20 |
| Water mixed with the above salts, | 0·72 |
| Sub-phosphate of lime, phosphate of magnesia, with traces of iron and manganese, | 77·50 |
| Silicic acid, | 0·70 |
| Hairs of caryopsis of oats, | 14·00 |
| | 100· |

Other portions of the concretion were found to contain a small amount of carbonate of lime. No biliary matter could be found in any part.

Before concluding this article, we may also allude to a very interesting specimen of a pedunculated calculus of the bladder. It was discovered in the body of an old woman, of whose previous history nothing was known. The peduncle and the nucleus of the calculus was a fibrous polypus growing from the upper and back part of the bladder; the peduncle from which the calculus was suspended was half turned upon its axis. The base of the calculus was broken off, the fragments probably having passed off by the urethra. What remained was nearly three inches in its longest, and two inches in its broadest diameter, and presented an elliptical shape. It nearly half filled the bladder, which exhibited considerable thickening in its muscular and mucous coats.—*Ibid. Oct.*, 1856.

4. Pericarditis. By *H. Bamberger.*—Fifty-seven cases of pericarditis, which occurred under the eye of Professor Bamberger, and of which twenty-seven proved fatal, are here analyzed, with a view of determining the etiological relations of this disease. The list only includes cases of undoubted pericarditis observed by the author himself, while all cases are omitted in which traces of former pericardial inflammation were discovered after death. The first point which results from the analysis is the infrequency of isolated pericarditis, which only occurred three times, or in about five per cent., and it is to be observed these cases were not fatal; in several of the fatal cases which were, during life, regarded as simple pericarditis, the post-mortem exhibited morbid conditions of other organs, which were nesessarily primary to the heart affection. Professor Bamberger's observations confirm the fact of rheumatism being the most frequent cause of pericarditis; his tables yield 17 cases dependent upon that disease, or 30 per cent.,—a ratio that approaches nearer to that established by Dr. Taylor than that by Dr. Chambers. The former found rheumatism to be the cause of 21 out of 40 cases, or 52 per cent.; the latter only found 18 out of 135 cases of pericarditis dependent upon rheumatism, or 14 per cent. The period of the supervention of the pericardial affection varied from the fourth day to four weeks after the commencement of the rheumatism; the majority of cases occurred between the sixth and fourteenth days. Next to rheumatism, Professor Bamberger finds tubercular disease to be the most frequent cause of pericarditis; it coexisted in 12 out of the 57 cases; and if we subtract 4 cases in which other complications, as aneurism and granular kidney, coexisted, there still remain 8 cases, or 14 per cent., in which tuberculosis was the undoubted cause of the pericarditis.

Professor Bamberger found 4 cases in which there was aneurism of the aorta, a circumstance at variance with Dr. Stokes's remark, that this complication is extremely rare. He is also opposed to the views of Dr. Taylor, regarding the frequency of granular kidney as a complication of pericarditis, as he only met with it in 7 cases, 4 of which presented other important complications, viz., 2 of tuberculosis, and 2 of cardiac hypertrophy. He only met with 1 case of granular metastatic pericarditis dependent upon pyæmia after traumatic inflammation of the knee-joint. One other case of pericarditis with puerperal fever might be explained in the same way. Professor Bamberger has only once observed pericarditis in connection with typhus. He adverts to the occurrence of pericarditis after scarlet fever, but does not appear to have observed it himself. The author briefly adverts to the symptomatology and therapeutics of pericarditis. With regard to the latter, we would only note the fact, that he altogether eschews mercury; not one of his patients received it; a circumstance which deserves the serious consideration of those practitioners who place their main reliance upon that drug, since the results obtained by Professor Bamberger are by no means despicable. His treatment consisted in the local application of leeches, and of the administration of digitalis, aqua lauro cerasi, acidum hydrocyanicum, bitartrate of potass, acetate of potass, and the like.

The article on valvular disease is based upon the analyses of 211 cases, observed by the author himself, 69 of which were watched up to the time of death. Cases of recent endocarditis are excluded, while only such cases of valvular disease are admitted in which there was insufficiency or contraction of the orifice. The following is the summary of the analysis:—Of the 211 patients, 109 were males, 102 female; of the 69 that died, 32 were male, 37 female. The valves were diseased as follows:

| | | |
|---|---|---|
| Mitral alone, . . . . . . | 58 males, . . . | 79 females. |
| Aortic alone, . . . . . . | 34 " . . . | 11 " |
| Pulmonary alone, . . . . . | 2 " | |
| Tricuspid, . . . . . . | 1 " | |

Total of single valves affected: 185 cases.

| | | |
|---|---|---|
| Mitral and tricuspid, . . . . | 5 males, . . . | 7 females. |
| Mitral and aortic, . . . . . | 6 " . . . | 3 " |
| Mitral, tricuspid, and aortic, . . | 3 " . . . | 2 " |

Total of complicated valvular affections: 26 cases.

The comparison of this analysis with the results obtained by the post-mortems is interesting; the latter showed 52 cases in which single valves were affected, viz.:

| | | |
|---|---|---|
| Mitral alone, . . . . . . | 9 males, . . . | 22 females. |
| Aortic alone, . . . . . . | 12 " . . . | 6 " |
| Pulmonary alone, . . . . . | 2 " | |
| Tricuspid alone, . . . . . | 1 " | |

The preponderance on the side of females in regard to mitral diseases, and of males in regard to aortic disease, is very palpable, and is further confirmed by what is observed in the complicated cases. Among the post-mortems 17 were complicated:

| | | |
|---|---|---|
| Mitral and tricuspid, . . . . | 4 males, . . . | 5 females. |
| Mitral and aortic, . . . . . | 2 " . . . | 2 " |
| Mitral, tricuspid, and aortic, . . | 2 " . . . | 2 " |

The analysis of the ages of the patients shows the greatest frequency of mitral disease to occur between the tenth and thirtieth years, and of aortic disease between the thirtieth and fiftieth. Acute articular rheumatism was ascertained to have preceded in 51 cases, or about 25 per cent. Professor Bamberger observes, that while his results correspond with those generally obtained by German observers, they differ from those obtained in England, where disease of the aortic valves is found to predominate considerably over mitral disease. It would appear that atheroma, which is the main cause of the former, is of more frequent occurrence in England than in Germany.

5. IDIOPATHIC TETANUS; TWO CASES, WITH ONE CASE AFTER ABORTION; AND NOTES ON TRAUMATIC TETANUS. By *Robert Annan*, Surgeon, Kinross.—At the recent trial of Palmer, few of the medical witnesses had seen *idiopathic* tetanus, and Sir Benjamin Brodie, and Mr. Blizard Curling, surgeon to the London Hospital, in particular, stated that death from idiopathic tetanus is rare in this country; the latter gentleman giving in evidence that he had seen more than fifty cases of *traumatic*, but not one of *idiopathic*, tetanus.

In a paper read before the Medico-Chirurgical Society of Glasgow (and reported in the Monthly Journal of Medical Science, Dec., 1845), Dr. Watson, senior physician to the Glasgow Royal Infirmary, remarks, "I have seen and treated a good many cases of *traumatic*, but never before one of *idiopathic* tetanus. Neither do I think such cases occur very frequently. In our hospital, this is only the third case since it was opened, fifty years ago; and though I may not have examined the Medical Journals very perfectly, to ascertain the experience of the profession on this point, the examination, so far as it has gone, confirms me in the above opinion. The cases narrated are not very numerous, and few of them well marked. Many, indeed, more nearly resemble hysteria than tetanus."

A reference to the Medical Journals, from the commencement of the present century, will, I believe, confirm the statement of Dr. Watson. In these circumstances, I send you the result of an experience here of about forty-four years, with a few incidental notes and cases. Previous, however, to my first *idiopathic* case, besides several examples of *traumatic* tetanus in the Peninsular war and in Kinross-shire, I had seen one case in a boy of fourteen, living near to Piershill Barracks, in the practice of my worthy master, the late Mr. J. H. Wishart, from blows from a carter's whip-shaft over the lumbar region; and in 1809 (being then a dresser), another case in the Royal Infirmary, under the care of Mr. (afterwards Sir William) Newbigging, in a lad of about twenty, from a slight puncture or scratch with a rusty nail, in the third joint of the little finger.* Both cases proved fatal about the end of the fifth or beginning of the sixth day. The first case of *idiopathic* tetanus was nearly as follows, occurring in 1828 or 1829:

*Case* 1.—John Hutton, cotton-weaver, Milnathort, æt. about 40, for some cause or other, removed for one afternoon, during damp weather, a linen or woollen apron, I forget which, used by operatives of this class. At first a slight chilliness was felt, which shortly afterwards was followed by rigors, and these again, in less than forty-eight hours, with stiffness of neck, and *trismus*, and general rigidity and contraction of the muscles of the trunk and both extremities, with a tendency to *opisthotonos;* death ensuing on the fifth day.

*Case* 2.—James Glass, æt. 28, foreman to Mr. Ireland, a farmer-proprietor on the western borders of Fifeshire, being in a state of profuse perspiration, incautiously put off his jacket and waistcoat, and during the two midday hours for dinner, so remained in the open air, the atmosphere being sultry and moist, after rather heavy rains. In less than two days he was seized with rigors, followed, as in Hutton's case, by the most decided symptoms of tetanus. He had been seen by Mr. White, a practitioner in Portmoak parish, before I was called to him,

* During this season, from the end of May to the middle of August, the weather being sultry, *hospital gangrene* very generally prevailed.

June 3, 1848, on the second day of the attack. On arrival, I found that a purgative had been given with moderate effect. The muscles of the jaw, neck, trunk, and both extremities, felt firm and contracted, the head and loins being drawn permanently backwards, and producing constriction over the respiratory organs. Pulse 90, and full. Countenance anxious, and indicating deep distress.

V. S. ad ℥xlvi; with no tendency to deliquium. Statim, Haust. cum. Tinct. Opii. ʒi. Calomel, gr. iij, et 2*da quaque hora*. h. s. Haust. cum. Tinct. Opii. ʒiss.

*June* 4.—No improvement, and constriction over chest increased. Emp. Lytt. sterno, et epigastrio. Calomel. Pulv. Ipecac. āā gr. iij. 3*tio quaque hora*. Vespere, et mane. Haust cum Tinct. Opii. ʒiss.

*June* 5 and 6.—Symptoms unabated, and he expired on the sixth day from the tetanic attack.

Being desirous of trying the effects of Ipecacuan and Calomel conjoined, I did not give the opiate to the extent that, perhaps, I might have done. And in a fatal *traumatic* case, in 1850, Tinct. Opii. was given, both by the mouth and as enema, so as very decidedly to *narcotize* the patient, a female of near sixty, with out any beneficial effects, save relief from pain. Since then, in a female of near sixty, attacked for the second time by exquisite *Angina Pectoris*, before my arrival Tinct. Opii. ʒij, and brandy had been taken. At the earnest request of the patient, an intelligent person, the Tinct. Opii. ʒij was repeated, Calomel gr. x, being added, and again repeated two several times without any visible effects; Tinct. Opii. ℥i, and Calomel gr. xxx, being thus taken within less than six hours without any effect; and powerful sinapisms being repeatedly applied, relief was obtained only after two bleedings of forty ounces each, within four hours. The blood, I may mention, at the end of two hours, saving a slight oozing from the surface of the coagulum, showed no proofs of serum. The patient was relieved, but slept none for twenty-four hours, no inconvenience being experienced from the opiate; a good example of the powers of the animal frame, *under spasmodic action*, in resisting the influence of narcotics. Two years after, this female had another similar attack, bleeding and leeching being conjoined. She is now in good health.

Mrs. B., æt. about 28, a farmer's wife. At the request of the relatives, I visited her along with Mr. Gall, surgeon, to whom, being first in attendance, I am indebted for *memoranda* of the case. Mrs. B. had previously borne four children, and, on 21st April, 1847, aborted in her fifth pregnancy, about the middle of the fifth month. The flooding was severe, but on 25th she got out of bed, and Mr. G.'s attendance was discontinued.

*May 5th*.—Mr. G.'s assistance was again required, stiffness of neck and threatenings of *trismus* having occurred on the preceding day, after having gone out of doors a very short distance, indeed, only a few yards. On 8th, my assistance was required, when all the symptoms of tetanus were completely developed. I saw her on the following day (9th), and calomel and opium having previously been freely given, partly as an *ultimum remedium*, and partly to move the bowels, which were obstinately costive, the tobacco injection was used with no good effects, and verifying the extreme caution required in the use of this powerful remedy. I then left her, in full expectation of death at an early period. She expired late on the evening of 10th, or very early on the morning of 11th May, her sufferings, notwithstanding the use of opium, in full doses, having been great.

Of cases of tetanus *after abortion*, Professor Simpson, in one of his most valuable "Contributions to Obstetric Pathology and Practice" (in Monthly Journal of Medical Science, February, 1854), has the following:—"In none of our modern obstetric books, nor in any of the various essays or works devoted to the consideration of abortion or puerperal diseases, is any illusion made, as far as I know, to the possible supervention of tetanus after miscarriage. It is a complication, however, which does occasionally take place; and it is always one very formidable in its character, and generally fatal in its issue." Two cases are then narrated similar to the above, with various others, all bearing on the same point. And I may here, in taking leave of this part of the subject, state that, having some months ago witnessed, in a fatal case of *traumatic* tetanus, at Milnathort, in a girl of seventeen years, the very marked relief from suffering afforded by the use of chloroform, I have often reflected that, had such means been then known, how much we should have been able to soothe, though it might not have cured the case of Mrs. B. and others.

In another case, the recovery from *traumatic* tetanus is worth noting.

Janet Dunn, æt. 6, a tall and slender girl, received from a female comrade, a blow on the dorsal region, with a shoemaker's hammer, followed by continued pain under the seat of the blow. After considerable suffering, at the end of eight days, *trismus*, followed by *lateral* tetanus or pleurosthotonos, ensued, but latterly ending in *opisthotonos*, so that she could be lifted from one position to another, like a curved deal board. She was then seen by Dr. Beveridge, late of E.I.C.S., and Mr. Fergusson, but both now deceased. In this state she lingered for thirteen days, great difficulty being experienced in getting down nourishment and cordials for support. At the end of thirteen days from the appearance of the tetanic symptoms, these rapidly abated on the discharge by the bowels of a large quantity of purulent matter, mixed with blood—no doubt, from an abscess near the spine, in its progress adhering to, and latterly opening into the intestine, and thus, perhaps, by counter-irritation and derivation, relieving the spinal irritation. This case occurred in September, 1820; the girl rapidly recovered, and is now alive, married, and the mother of several children.

In this case, the two things remarkable are, the occurrence of *pleurosthotonos*, a symptom of very rare occurrence, and the period of continuance of acute *traumatic* tetanus; as in this disease, experience shows that, in the great majority of cases, death generally takes place before the end of the fourth, or at most, fifth or sixth day.—*Edinburgh Med. Journal*, Nov., 1856.

6. ON THE THERAPEUTIC EFFECTS OF CHARCOAL IN EPIDEMICS OF MEASLES AND CHOLERA. Extracted from a Report to Government for the year 1854. By Dr. *Wilson*, Colonial Surgeon, New Plymouth, New Zealand.—Throughout the course of the epidemic (measles), I never observed diarrhœa to be beneficially critical, but otherwise; and as invariably induced either by imprudent exposure in the early stages of the disease, by sudden check to perspiration; or, when the state of the patient, or unavoidable circumstances, came in the way of promoting a perspirable state of skin. Hence, I never hesitated to check the laxity by such correctives as had also a sudorific tendency; and of these I found none so efficient as the ordinary wood *charcoal* in powder, though, had the prepared sort been within my reach, I should have preferred it as of more efficacy.

In the means of cure employed there was no novelty, excepting, as stated, the general substitution of *charcoal* for other remedies in those cases where diarrhœa supervened, and became the prominent object of attention; and that, when assiduously and sufficiently exhibited, never failed, within my knowledge, to have a promptly beneficial effect; and thus, being a medicine which I have often employed for many years past, occasionally extensively, and never without becoming more and more awakened to its importance as a remedial agent, I trust I may be excused if I occupy a space of this report by a cursory notice regarding it.

My attention was first drawn thereto by observing its powerfully deodorizing and cleansing properties, as an external application in fetid and gangrenous ulcers, but more especially, afterwards, by some observations of the late venerable Dr. Robert Jackson, in his "*Sketch of Febrile Diseases*," as regards its internal administration in dysentery, and allied affections of the alimentary canal.

Following in the footsteps of that very experienced physician, my first trials of *charcoal* as an internal remedy were in cases of simple acute dysentery; though I would observe that, as in the then sphere of my practice, dysentery was not a disease of frequent occurrence, my opportunities of testing the powers of the remedy were too limited to do more than to satisfy myself that there was no exaggeration in the Doctor's statements. So far, indeed, from this, that, as his experience had foretold, I found the result of its administration, in almost every case, the echo of his own words—"soothing and pleasant beyond the effect of common remedies, the excess of the evacuations becoming not only restrained, but the matter changed from blood and mucus, putrid and offensive, to figured feculence."

As illustrative of the words now quoted, I shall give here the short statement of a case to which I was called in consultation nearly twenty years ago. The patient was the child of the British consul at Port St. Mary, in Spain, was about five years of age, and had been under the influence of the disease two or three days before my visit. The family physician was a Spaniard of the *medicine expectante* species, and so leniently *Broussaian*, that he had confined his treatment o the disease to the application of a couple of leeches, and the ordering of a ptisan, composed of a solution of gum-arabic in an infusion of the blossoms of the common mallow, and of three other equally simple wild-flowers, which, together, and from time immemorial, over all the Peninsula, have maintained the pre-eminent designation of "*Las cuatro flores cardinales*," but which, according to my experience —and I drank the infusion plentifully, on one occasion, for months—has been acquired more likely from a harmless placebo quality, than from any curative virtue they have thus nominally got credit for. The father, an intelligent Englishman, had had the prudence, on the day of attack, to give the child a dose of castor oil; but, notwithstanding, the disease, at the time I saw the patient, was still crescent, face greatly flushed, fever high, tormina severe, and tenesmus urgently frequent; dejections, mucus and blood, and coming off in a very frothy state. He had not slept for nearly twenty-four hours, and in every way great distress was indicated. My prescription was novel, too, and did not meet the approval of the medical attendant; but, as I had the confidence of the parents, he did not persist in opposition. Accordingly, a tablespoonful of common levigated *charcoal* was rubbed up with the white of an egg, and these, being diffused in eight or ten ounces of chicken broth, the mixture was exhibited per anum. It was returned almost immediately, but a second, which followed so soon as it could be prepared, was retained, and now, in or less than five minutes the patient dozed off into a quiet, sound sleep, which continued uninterrupted for the space fully of five hours. Thus, as Dr. Jackson observes, in relating a similar case, "the relief was instant and perfect," the fever promptly abated, and, in short, I may sum up that the disease was at once arrested, thenceforward requiring, in ulterior treatment, only one or two laxative doses of castor oil, to perfect the cure.

But previously, by some years, to the occurrence of the case above narrated, I was induced, partly from circumstances adverted to in the report, to test the powers of *charcoal* as a prophylactic in yellow fever, at the outset of a very active epidemic of that disease, which occurred at Gibraltar, in the autumn of 1828. The experiments, instituted chiefly from appearances observed on *post-mortem* examination, were almost entirely confined to my own person; and these impressed on my mind a conviction that, though I was not exempted from attack, yet the mildness of that,—for it hindered me only one day from my hospital duty,—might properly be imputed to its influence. For, at the period thereof, namely, the first phasis of the epidemic's career, mild grades were not common; and my temperament—ardent, sanguineous—was, for such, unfavorable. Moreover, few were more exposed to the morbid cause, whether considered as infectious or as contagious; or to the predisposing causes, viz., fatigue, care, anxiety, and, no doubt, a common share of the prevailing panic. Yet many, greatly more favorably circumstanced, went emergently through, or fatally succumbed to, severe grades. I had no experience of it as a remedy in the developed disease, for I found patients, very probably from associating it with the black vomit, invariably averse to its administration; and as, in the after-progress of the epidemic, medical men had but little time to throw away in endeavors to overcome repugnances, or to note with attention the results of novelties, I was neither persevering nor general in my attempts. But I would observe, as discriminatory from dysentery, and from what the numerous autopsies demonstrated as to the state of the alimentary canal, that it is less to be depended on in this disease in the form of enema, than as administered in large doses, and frequently, by the mouth.

Until the summer of 1834, no opportunity offered to me of researching further as to the worth of *charcoal* administered internally. But, in that season, I happened to be a sojourner in that city of Andalusia, called Jerez, at the time it became subjected to a violent outburst and overspread of cholera—a disease, I may remark, I had rather longed to see in its epidemic form, and in that fierce instance of its workings, I may verily say, I was greatly more than gratified.

I was not, at the time of the occurrence, residing in Jerez in a professional capacity; but, under the strange circumstance that some of its *medicos*, early in the epidemic, *absconded* therefrom, and that, to meet the dire exigency of the visitation, not the tithe of an adequate number remained behind, it was no time to be simply a looker on. Accordingly, I proffered my services to the Government authorities, and an hospital being put under my charge, I had now, and in all ways, ample opportunity of acquiring some knowledge of the disease. By a reference, then, to my notes of that period, I may here introduce some detached remarks therefrom, relative to the use of *charcoal* in it, whether employed as a prophylactic or as a remedial agent.

"In the early part of the first stage, where there is only some anomalous feeling of *malaise,* attended with slight laxity of bowels, and which has obtained among the Spaniards the name of *colerina* or little cholera, a few doses of Lavitz's *prepared charcoal,* both by the mouth and by enema, and the exhibition on the succeeding day of an oil laxative, and living cautiously for a few days, are sufficient means, generally, to check the farther advance of the disease.

"Throughout the first stage of formal attack, when I could prevail on my patients to take *charcoal,* it was administered; but many objected to take it by the mouth, and it was not with the multitude I could insist, since I could not compel obedience, neither give time to superintend its necessarily frequent administration, nor place reliance on the quantity or *quality* given. From repeated experiments on myself and others, I am well assured that the latter is of considerable consequence, the remedial power being much deteriorated by exposure to the air. Therefore, it should not only be very carefully prepared, but be kept for use in accurately stoppered bottles or phials. I would farther observe that, hitherto, I have always used the charcoal prepared from the olive-root, which is a compact, hard wood—not, however, from choice, but merely from its being that which most conveniently offered. But I consider it as not improbable that experience may detect a difference of medicinal quality in opposite sorts. Thus we see gunpowder manufacturers adopt, as giving strength to their compound, charcoal made from the willow and such like light woods. Hence, we may argue that, if levity indicates, in the composition of that, a superiorly energizing effect, so may the charcoal of the willow, cork, or sponge, be found to be superiorly efficacious, as a medicine, to that prepared from the heavy woods.

As a prophylactic, after the publication of some instructions to the people, the charcoal gained a speedy and extraordinary reputation; and some of the blacksmiths, who got from me the mode of preparing it in large crucibles, acknowledged to the gain of seven and eight dollars a day by retailing it in small quantities to the multitudes of applicants. But, without having once tried whether it had or had not virtues, all my *confreres* were doggedly opposed to its administration; and much of the people, in some degree influenced, but greatly more from the novel, yet homely character of the article, as also from the required largeness and frequency of the dose, could not readily be brought to rely confidently on it as a remedial agent in so hurrying a disease. Ultimately, therefore, when, from the general explosion of the epidemic over the city, I was obliged to post from patient to patient, or rather from house to house, and to square, and lane, and street, it was only in very occasional cases that I could continue its use by the mouth. But from first to last, I persevered with, and met no balking to, its employment in the form of glyster. But it was given also, in numerous cases, by the mouth, and with such general good effect, as to have impressed me with the firm conviction that, in all stages of the disease, it is a most beneficial adjuvant, and anterior to the asphyxial stage, and in that of reaction, most eminently curative.

It may possess a specific febrifuge power or quality, but I am disposed to ascribe its virtues to its general antacid and absorbent property, and no less to its controlling and corrective power over all ferments—vinous, ascescent, and putrefactive. Hence, as it was my belief that foul, morbid fermentation in the stomach and bowels, was one of the most constant symptoms, and not the least of aggravating causes in the progression of the disease, I could see nothing more likely to correct and mitigate it than the agency of this medicine, and more so, as I found that, by restricting my patients to the use of those articles the least liable to run into the fermentative process, as *e.g.* rice water, linseed tea, and unleavened cakes or biscuit, were the most likely to effect a cure: while, on the other hand, inattention to such dieting, or deviation therefrom, were the most certain means of both accelerating and aggravating the disease.

Generally speaking, Spaniards are very averse to *post-mortem* examinations; but the hospital which I superintended, together with, at that sad time, the almost utter regardlessness of the living as to what became of the dead, afforded me every facility; and I availed myself thereof as often as my professional avocations would permit. And in corroboration of the opinion that a fœtid, fermentative state of the intestinal contents has greatly to do, if not in promoting, certainly in aggravating, the disease, I can affirm that, invariably, I found these, notwithstanding the gallons of watery fluid that had passed off during the course of the disease, most offensively feculent, and not unfrequently remarkably scybalous. I do not, of course, advance this last circumstance as extraordinary, supposing, as we may, that the fluids of the system poured into the alimentary canal, acted thereon simply as saline purgatives often do in dropsies, in the autopsies of which disease we often find impactions of scybalæ of very old date. Thus, in such, on one occasion, I was present at the detection of the seeds of the prickly pear, which, it was accurately ascertained, had been eaten six months before, notwithstanding that saline purgatives had repeatedly been used intermediately. But the important inference to be drawn from the circumstance of the retention of putrid feculent matter, and viewing this as the pabulum, is the necessity that exists of administering oily laxatives during the stage of reaction, or, at latest, so soon as that begins to abate; for I found, until I adopted this plan of treatment, that my cases of relapse were frequent, and not unusually fatal. But, what experience and *post-mortem* autopsy proved to me, could not be made alike obvious to my patients. Accordingly, I met with much, and very often fatally-ending opposition to this evacuating practice, as they could not be made to comprehend how a disease of so untowardly diarrhœtic a character, was to be otherwise than deteriorated by the administration of laxatives.

I have never had an opportunity of using *bone charcoal* internally, but, from its ascertained superior powers as a deodorizer, I consider that it deserves to be tested by experiment in such affections as I have here indicated.

I may add, in conclusion of this short notice regarding charcoal, that during the course of the Jerez epidemic now observed on, I was affected repeatedly with premonitory symptoms, and once to the degree of what, as already observed, was denominated by the Spaniards *colerina*. But, on every one of these occasions,

I derived almost immediate relief from a dose or two thereof; and thus, no doubt, I escaped the endurance of a more formal attack.

Taken daily as a prophylactic, the only inconveniences complained of were its sudorific and constipating effects.

In gastrodynia, when ascescency is a prominent symptom, it gives, though only temporary, very prompt relief.

